# Complementary DNA/RNA-Based Profiling: Characterization of Corrosive Microbial Communities and Their Functional Profiles in an Oil Production Facility

**DOI:** 10.3389/fmicb.2019.02587

**Published:** 2019-11-07

**Authors:** Silvia J. Salgar-Chaparro, Laura L. Machuca

**Affiliations:** Curtin Corrosion Centre, WA School of Mines: Minerals, Energy and Chemical Engineering, Curtin University, Perth, WA, Australia

**Keywords:** DNA, RNA, 16S rRNA gene, oil production, produced water, injection water, microbiologically influenced corrosion (MIC)

## Abstract

DNA and RNA-based sequencing of the 16S rRNA gene and transcripts were used to assess the phylogenetic diversity of microbial communities at assets experiencing corrosion in an oil production facility. The complementary methodological approach, coupled with extensive bioinformatics analysis, allowed to visualize differences between the total and potentially active communities present in several locations of the production facility. According to the results, taxa indicative for thermophiles and oil-degrading microorganisms decreased their relative abundances in the active communities, whereas sulfate reducing bacteria and methanogens had the opposite pattern. The differences in the diversity profile between total and active communities had an effect on the microbial functional capability predicted from the 16S rRNA sequences. Primarily, genes involved in methane metabolism were enriched in the RNA-based sequencing approach. Comparative analysis of microbial communities in the produced water, injection water and deposits in the pipelines showed that deposits host more individual species than other sample sources in the facility. Similarities in the number of cells and microbial profiles of active communities in biocide treated and untreated sampling locations suggested that the treatment was ineffective at controlling the growth of microbial populations with a known corrosive metabolism. Differences in the results between DNA and RNA-based profiling demonstrated that DNA results alone can lead to the underestimation of active members in the community, highlighting the importance of using a complementary approach to obtain a broad general overview not only of total and active members but also in the predicted functionality.

## Introduction

Corrosion refers to the deterioration of metals that results from its interaction with the environment. It is a natural process that affects several sectors such as production, transportation and refining of hydrocarbons (Hansson, 2011). This phenomenon generates millions of dollar losses to the world’s industry every year (Franklin and White, 1991; Kip and Van Veen, 2015). In fact, the latest estimation of the global corrosion costs quantified as US$2.5 trillion, without including safety or environmental consequences (Koch et al., 2016). From this total cost, microbiologically influenced corrosion (MIC) accounts for almost 20% of external, and 40% of internal corrosion problems in pipelines (Wolodko et al., 2018). MIC is known as the deterioration of metals that results from the presence and activity of microorganisms on their surfaces (Beech and Sunner, 2004). It was first identified in 1963 (Booth and Mercer, 1963), but its significance was not commonly recognized in the same decade (Usher et al., 2014). Nowadays, it is well known that the participation of microorganisms in the corrosion process can significantly increase the corrosion rates, representing a big concern to the integrity of industrial infrastructure, particularly oil and gas facilities (Usher et al., 2014; Xu et al., 2016).

Microorganisms change the electrochemical conditions at the metal/solution interface by the attachment of cells, biofilm formation, and subsequent release of metabolites, which induces or accelerates the corrosion process (Moura et al., 2013). MIC is characterized by a particular morphology of damage – localized pitting (Little and Lee, 2007), with corrosion rates reported up to 10 millimeters per year (Machuca and Polomka, 2018). Remarkably, MIC is not constrained to a unique corrosion mechanism (Lewandowski and Beyenal, 2009; Kakooei et al., 2012; Urquidi-Macdonald and Macdonald, 2014; Li et al., 2018). The main mechanisms described for MIC include the formation of concentration cells, the production of corrosive metabolites, the removal of protective films, and the production of unprotective surface layers (Skovhus et al., 2017). Lately, MIC has been reclassified into two different mechanisms, chemical MIC (CMIC) that considers metal deterioration induced by corrosive chemical species produced via microbial metabolic activity (indirect corrosion), and electrical MIC (EMIC) that refers to the damage caused by direct microbial uptake of electrons from the steel (direct corrosion) (Enning and Garrelfs, 2014). Causative microorganisms have been classified in microbial groups according to their metabolic activities, such as sulfide producing prokaryotes that include sulfate and thiosulphate reducers (Machuca et al., 2017; Machuca and Polomka, 2018), acid-producing (Gu and Galicia, 2012; Gu, 2014), methanogens (Uchiyama et al., 2010), iron-oxidizing (Ashassi-Sorkhabi et al., 2012; Liu et al., 2014), and iron-reducing bacteria (Herrera and Videla, 2009). Microorganisms with these metabolic capabilities are part of the normal microbiota of petroleum reservoirs (Magot et al., 2000; Ollivier and Magot, 2005). Microbial populations in oil reservoirs can reach the surface and colonize the metal infrastructure of the production facilities during the oil and gas extraction process.

Monitoring microbial activity in production facilities is part of the corrosion management of oil and gas industry assets. Microbiological assessment is routinely performed to detect the presence of MIC causative microorganisms and to evaluate the effectiveness of biocide treatments used to mitigate against MIC. Traditionally, culture-based techniques have been used to identify the presence of known corrosive microbial groups in industrial facilities (Muyzer and Marty, 2014; Beale et al., 2016; Machuca and Polomka, 2018). Since culture media cannot recover all microorganisms present in the environment (Keasler et al., 2012; Chakraborty et al., 2014), culture-independent techniques are used to complement cultivation based analyses. In the last decades, molecular microbiological methods have been implemented to improve the understanding of the microbial ecology of a system (Chakraborty et al., 2014). Within molecular methods, 16S rRNA gene amplicon sequencing is the most implemented method to study the biodiversity of oilfield environments (Lin et al., 2014; Wang et al., 2014; Li et al., 2017a; Okoro and Amund, 2018). Despite the disadvantage in the data interpretation due to the variation of 16S gene copy number among species (Crosby and Criddle, 2003; Acinas et al., 2004), the use of this sequencing approach on the biofilm communities recovered from corroded metals has allowed for taxonomic identification of microorganisms likely to be associated with corrosion failures (Vigneron et al., 2016, 2018; Li et al., 2017a). Considering that DNA-based analysis cannot discriminate between active and inactive species, RNA-based analyses have become popular in microbial ecology investigations as an alternative methodological approach to generate information of active members in the communities of different environments (Moeseneder et al., 2005; Bastias et al., 2007; Lillis et al., 2009; Kim et al., 2013). Nevertheless, the suitability of amplicon sequencing of 16S rRNA transcripts for identifying the active microbial populations that may be involved in corrosion of oil production systems has rarely, if ever, been explicitly addressed.

This work aimed to determine whether the application of complementary analysis using amplicon sequencing of the 16S rRNA gene and transcripts would provide relevant information on the microbial communities recovered from an oil production facility with corrosion issues. The production facility chosen for this investigation has exhibited several incidents of pinhole leaks or rapid reduction in wall thickness in pipes and vessels in the last decade. To the present, the causes of the increase in the corrosion rates of assets at the facility are uncertain. Corrosion processes were previously attributed to the presence of deposits called “schmoo,” which are a combination of oil, corrosion inhibitor, produced fines and scales (O’Reilly et al., 2016). The formation of this material in the pipe walls reduces the effectiveness of corrosion inhibitors, increasing the risk of corrosion failures. However, the localized corrosion attack evidenced in the facility and the detection of microorganisms previously associated with MIC has raised the concern that microbial activity might also play a part in these corrosion failures. Including the RNA-based sequencing approach in the microbiological assessment of the oil facility helped identify active members in the community. In addition, the comparison of both methodologies through several bioinformatics tools allowed to visualize differences in the profiles between total and active communities, as well as the effect of environmental parameters (local operational conditions) and biocide treatment on their composition. Functional profiles from 16S rRNA data were predicted as a complementary and cost-effective metagenomic pre-study for identifying the metabolic capabilities of the oilfield communities, and to relate potential differences in the DNA and RNA-based results with predicted functionality.

## Materials and Methods

### Site Description and Sampling

**FIGURE 1:**
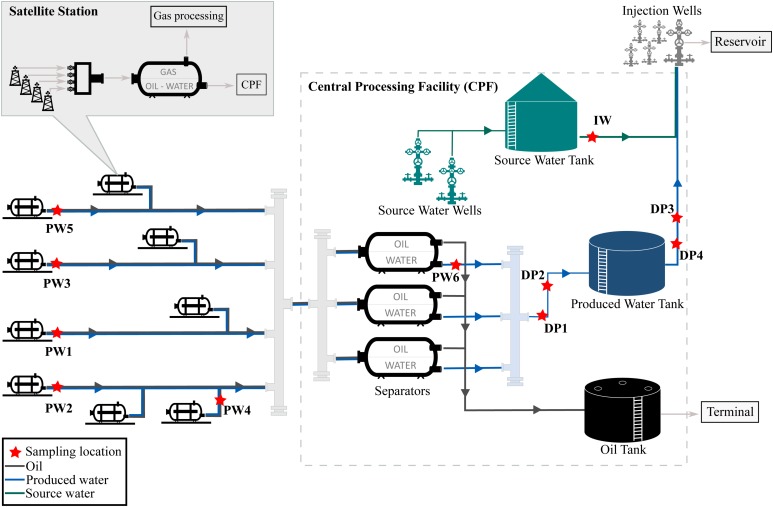
Process flow diagram of the oil production facility.

The oil production facility from which samples were taken is located on the north-west coast of Western Australia. The oilfield has been operating for many years and uses water flooding to increase reservoir pressure and thereby stimulate production. Approximately 80,000 barrels of water per day (BWPD) are injected into the reservoir from 268 injector wells. Water used for this practice consists of a mixture of source water extracted from source water wells, and recycled produced water, also known as produced water re-injection (PWRI). Produced fluids extracted by the oil producing wells are transported to satellite stations distributed in the facility, where a biphasic separation is carried out by degasser vessels. Then, the water-oil mixture is transported to the central processing facility (CPF) for further separation of oil and water in low-pressure separator units. After separation, oil is shipped out of the facility and produced water is reinjected into the reservoir. A schematic diagram of the production facility is presented in Figure 1.

The assessment of the microbial composition of total and potentially active communities in the facility was carried out by collecting six (6) produced water samples, four (4) deposits samples (schmoo), and one (1) injection water sample. Produced water samples were collected from five (5) satellite stations downstream of the degasser facility and one (1) after the oil-water separator at the CPF. Each satellite station recovers fluid from different oil production wells, while the CPF water sample comingles fluids recovered from the satellite stations in the oilfield. Deposits samples accumulated at 6 o’clock position were collected from four different locations of the pipeline in the produced water recovery system. Deposits were sampled during replacement activities of corroded pipeline downstream the low-pressure separators. All replaced pipeline was covered by approximately 3 cm of schmoo material; only one of the samples was collected in an exact indication of wall thickness loss. The injection water sample was collected from the storage tank, downstream the source water wells. Samples were coded according to the source (produced water = PW, deposits = DP, injection water = IW). Sampling locations are indicated in Figure 1. Samples PW1 and PW2 belong to stations with biocide treatment tetrakis (hydroxymethyl) phosphonium chloride (THPS) whereas the other stations were not under chemical treatment. Produced water is also treated with Acrolein (ion dissolver and biocide) in the CPF separators before entering the produced water recovery system. In this way, PW6 and all deposits are biocide treated samples. Sample DP3 was the only sample collected in an exact indication of wall thickness loss.

Water samples were collected in baked and autoclaved glass containers, after 1 min of line flushing, whereas deposits samples were collected using sterile spatulas and placed in DNase/RNase free sterile plastic containers. Six samples were collected from each sampling location and processed separately to obtain triplicates for the DNA and RNA analysis, respectively. All containers were tightly sealed to avoid oxygen intrusion and immediately transported to the oilfield production laboratory for processing and preservation within maximum 30 min after collection. Oil-water samples were decanted in sterile separatory funnels for oil phase removal, and 500 mL of water were filtered through sterile 0.2 μm pore size membranes to harvest microbial cells. Deposits and filter membranes were immersed in RNAprotect^®^ Bacteria Reagent (QIAGEN) and transported at 4°C to a research facility (2 days after collection) for further processing. Upon arrival, RNAprotect was washed from the samples with diethyl pyrocarbonate (DEPC) treated water, and samples were stored at −80°C until DNA/RNA extractions were conducted (maximum 1 week upon collection).

### Chemical Characterization

Chemical analysis of produced water and deposits (performed by a certified commercial laboratory) were carried out following US EPA, APHA (American Public Health Association, 2005), and in-house test methods. Analyses included: (a) pH (Thermo Scientific, Orion Star A329 pH probe and meter); (b) conductivity (Thermo Scientific Orion 5-Star Conductivity meter); (c) total petroleum hydrocarbons (TPH) by Gas Chromatography-Flame Ionization Detector (GC-FID) (US EPA 3510C); (d) volatile fatty acids (VFA) by High Performance Liquid Chromatography (HPLC) (in-house method); (e) metals Fe, Mg, Na, K, Ca by atomic absorption spectroscopy (AAS) (APHA 3030 and 3110) and S, Cr, Cu, Mn, Ni, Zn by inductively coupled plasma optical emission spectrometry (ICP- OES) (APHA 3030 and 3120); (f) total nitrogen, total phosphorus, nitrate-N, chloride and sulfate measured using an automated Colorimetric/Turbidimetric Aquakem System (APHA 4500); (g) thiosulphate measured using an in-house method involving standardized iodate/iodide titration following by formaldehyde pre-treatment; (h) total organic carbon (TOC) by the high-temperature combustion method (APHA 5310B), and (i) alkalinity by titration (APHA 2320).

### Microbial Enumeration

The number of cultivable sulfide-producing prokaryotes (SPP), acid-producing bacteria (APB), iron-reducing bacteria (IRB), and iron-oxidizing bacteria (IOB) were determined by the serial dilution method described in the standard test method NACE TM0194 (NACE International, 2014), which is the most widely used technique in the industry for monitoring oil field microbes. For counting SPP microorganisms, a culture media described elsewhere was used (Suarez et al., 2019a). Other microbial populations were evaluated using culture media suggested in the standard mentioned above. The serial dilution method consists of preparing 10-fold dilutions of the sample into liquid media. For this, 1 mL of water or 1 g of deposits was inoculated in a glass vial with 9 mL of culture medium and diluted six times (10^6^). Each serial dilution was conducted in duplicate. Culture vials were incubated at the temperature found *in situ* during sample collection (40°C), for a total of 28 days. Positive growth was determined by changes in the culture media as per NACE TM0194 guidelines.

### Nucleic Acids Extraction

To assess the potential involvement of the microbial communities in the corrosion failures experienced in the oil production facility, DNA and RNA-based profiling were used for the molecular characterization of the total and potentially active microorganisms, respectively. DNA was extracted from water samples using the DNeasy PowerWater Kit (QIAGEN) according to the manufacturer’s instructions with the following modification: filters were placed into the PowerWater DNA Bead Tube containing solution PW1 and heated at 65°C for 10 min before the Vortex step. RNA was extracted using the RNeasy PowerWater Kit (QIAGEN) following the manufacturer’s instructions. DNA and RNA concentrations were quantified fluorometrically with the Qubit dsDNA and RNA HS Assay kits (Life Technologies). Afterward, total RNA was treated with DNase I (Thermo Scientific) to remove remaining DNA. To verify the complete removal of DNA, a PCR reaction targeting the 16S rRNA gene was performed. Subsequently, RNA was purified and concentrated by using the RNeasy MinElute Cleanup kit (QIAGEN). Purified RNA was converted to cDNA by using the QuantiTect Reverse Transcription kit (QIAGEN). DNA and RNA from deposits were extracted as mentioned for water samples but employing the DNeasy PowerSoil and RNeasy PowerSoil Kits (QIAGEN), respectively. Despite several attempts with modified conditions, the extraction of high-quality RNA from the IW sample or cDNA synthesis from DP1 – DP4 samples failed.

### Library Preparation and Sequencing

Polymerase chain reaction (PCR) and sequencing were performed by the Australian Genome Research Facility. PCR amplicons were generated using the primers 341F (5′ CCTAYGGGRBGCASCAG 3′) and 806R (5′ GGACTACNNGGGTATCTAAT 3′) (Yu et al., 2005). Thermocycling was completed with an Applied Biosystem 384 Veriti and using AmpliTaq Gold 360 master mix (Life Technologies, Australia) for the primary PCR. The first stage PCR was cleaned using magnetic beads, and samples were visualized on 2% Sybr Egel (Thermo-Fisher). A secondary PCR to index the amplicons was performed with TaKaRa Taq DNA Polymerase (Clontech). The resulting amplicons were cleaned again using magnetic beads, quantified by fluorometry (Promega Quantifluor) and normalized. The equimolar pool was cleaned a final time using magnetic beads to concentrate the pool and then measured using a High-Sensitivity D1000 Tape on an Agilent 2200 TapeStation. The pool was diluted to 5 nM and molarity was confirmed again using a High-Sensitivity D1000 Tape. This was followed by sequencing on an Illumina MiSeq instrument with a V3 (600 cycles) kit (Illumina).

### Bioinformatics and Statistical Analysis

The Quantitative Insights Into Microbial Ecology Software (QIIME, v1.9.1) (Caporaso et al., 2010) was used for the analyses of the 16S rRNA gene sequences generated with the Illumina MiSeq. Paired-end reads were assembled by aligning the forward and reverse reads using PEAR (v0.9.10 - 64 bit) (Zhang et al., 2014) with default parameters. Then, Primers were identified and trimmed with Cutadapt (v1.10) (Martin, 2011) using default settings. Afterward, USEARCH (v10.2) (Edgar, 2010) was used for quality filtering, dereplication, denoising, and clustering into zero-radius operational taxonomic units (zOTUs) with the UNOISE3 algorithm. Chimeric sequences were removed using UCHIME (Edgar et al., 2011) with SILVA as reference database (SILVA v132) (Yilmaz et al., 2014). Filtered sequences were mapped to chimera-free OTUs, and the zOTU table was created using VSEARCH (v1.1.3) (Rognes et al., 2016). Taxonomic classification of the reference sequences (zOTUs) was performed by similarity searches using BLAST against the same SILVA database. Species richness, alpha and beta diversity estimates were determined using the QIIME algorithms. Sample comparisons were done at the same surveying effort, utilizing 34,532 by random selection.

Statistical analyses and graphs were conducted employing R (v3.4.3) (R Core Team, 2014), and PAST (v3) (Hammer et al., 2001) software. Results of statistical tests were considered significant with *p* ≤ 0.05. The statistical analyses implemented depended on the normality of the data in each variable. Shapiro–Wilk test (Shapiro and Wilk, 1965) was used to determine data distribution and homogeneity of variance. To test differences in variables with normal distribution we used analysis of variance (ANOVA) followed by Tukey’s multiple comparisons (Tukey, 1949). For those variables with a non-normal distribution, we used the Kruskal–Wallis test followed by Dunn’s multiple comparisons. The shared microbial zOTUs among communities in the produced water, injection water and deposits were investigated using “VennDiagram” R package (Boutros Paul and Chen, 2011). Relative abundances of specific microbial groups in the total and active communities were studied at phylum and order level, whereas differences in the microbial composition of all sample sources was investigated at genus level. Bart charts of the microbial communities with phylogenetic groups with relative abundances equal or greater to 1% in at least one sample were created using the “ggplot2” R package (Wickham, 2016).

To visualize the multivariate dispersion of the community composition a non-metric multidimensional scaling (NMDS) analysis was performed based on the Weighted UniFrac distance (Lozupone and Knight, 2005), lines for joining samples collected in the same sample source were projected onto the ordination, utilizing the function *ordiellipse*. The effect of environmental parameters on the microbial community was analyzed using the *envfit* function and projected into the ordination with arrows. *Ordiellipse* and *envfit* functions are contained in the “vegan” R package (Oksanen et al., 2015). Permutational analysis of variance (PERMANOVA) and analysis of similarities (ANOSIM) were used to test for significant differences in beta diversity, Bray–Curtis distance (Bray and Curtis, 1957) was used in these tests. To identify the microbial orders associated with the produced water at the different stations or within each sample source, an analysis based on the point biserial correlation coefficient was performed using *multipatt* in the “indicSpecies” R package (De Caceres and Legendre, 2009). For visualization, a network was generated using stations or sample source as source nodes, and the bacterial orders as target nodes. All taxa with significant associations were visualized in the networks. The network was performed using the edge-weighted spring embedded layout algorithm in Cytoscape (v3.5) (Shannon et al., 2003), with the edge weight corresponding to the association strength of each order with each sampling location.

The functional profile of the microbial communities was predicted using the “Tax4Fun” R package (Aßhauer et al., 2015). FTU (fraction of taxonomic units unexplained) values of the prediction were relatively low in most of the samples (FTU $\bar{\text{x}}$ 26) which indicated that the majority of the zOTUs were included in the functional prediction. Comparison of the functional profiles predicted from the DNA and RNA-based sequencing was performed using the average of the relative abundance predicted per pathway in all samples. Linear discriminant analysis (LDA) effect size was employed through LEfSe v1.0 (Segata et al., 2011) to identify KEGG pathways as significant biomarkers of the microbial communities and sample sources. For this analysis, the alpha parameter significance threshold for the Krushkal–Wallis (KW) test implemented among classes in LEfSe was set to 0.05 and the logarithmic LDA score cut-off was set to 2.0. All analyses were performed through the Galaxy server (Goecks et al., 2010).

## Results

### Chemical Characterization

The chemical composition of the water and deposit samples is shown in Tables 1, 2, respectively. A clear difference in the chemistry of produced water and injection water was evidenced. Injection water contained lower levels of petroleum hydrocarbons (TPH), organic carbon (TOC) and volatile fatty acids (VFAs), as well as less total dissolved solids as indicated by the conductivity. Produced water samples exhibited similar characteristics in the different sampling locations, pH close to neutrality, high salinity, and similar content of organic compounds. The main difference in the chemical composition of produced water samples was the concentration of metals. PW1, PW2, and PW5 reported higher levels of iron and manganese. Additionally, PW2 displayed higher levels of zinc, as well as the presence of sulfate. Chromium, copper, nickel, sulfur, and thiosulphate were not detected in any of the water samples. On the other hand, deposits samples exhibited more variation in chemical composition among them. Different to produced water, deposits exhibited high levels of sulfur, sulfate and thiosulphate. Samples also contained high levels of petroleum hydrocarbons and metals such as iron and manganese, the last two probably associated with under-deposit corrosion (UDC). Nitrogen oxides, nitrate, and nitrite were not detected in any of the samples.

**TABLE 1:** Chemical composition of produced water and injection water samples.

**Compound/element^a^**	**LOD^b^**	**Produced water**						**Injection water**
			
		**Stations**					**CPF**	**Tank**
				
		**PW1**	**PW2**	**PW3**	**PW4**	**PW5**	**PW6**	**IW**
pH	–	6.75	6.95	7.34	7.18	7.03	7.02	6.92
Conductivity (mS/cm)	–	60.1	60.3	68.3	65.7	65.2	59.2	48.0
TPH^*c*^ C6-9 (mg/L)	0.02	2.2	0.64	1.1	2.3	44	4.4	<0.02
TPH C10-14 (mg/L)	0.02	27	8.7	10	25	210	43	0.74
TPH C15-28 (mg/L)	0.04	31	9.4	9.5	31	200	44	0.17
TPH C29-36 (mg/L)	0.04	1.7	0.28	0.19	3.0	20	2.6	<0.04
TPH C > 36 (mg/L)	0.04	0.29	0.08	<0.04	0.30	5.4	0.34	<0.04
Calcium (mg/L)	0.1	390	530	570	610	750	490	500
Iron (mg/L)	0.01	10	11	4.0	3.7	12	4.9	0.55
Potassium (mg/L)	0.1	190	370	170	160	170	170	310
Magnesium (mg/L)	0.1	190	270	370	310	370	240	140
Manganese (mg/L)	0.01	0.16	0.15	0.06	0.08	0.15	0.07	0.07
Sodium (mg/L)	0.1	13,000	13,000	15,000	14,000	14,000	14,000	9,800
Zinc (mg/L)	0.01	0.16	1.7	<0.01	0.30	0.04	<0.01	0.07
Ammonia-*N* (mg/L)	0.02	45	42	52	47	47	43	41
Chloride (mg/L)	5	22,000	23,000	28,000	27,000	26,000	25,000	20,000
Sulfate (mg/L)	1	<1	2	<1	<1	<1	<1	<1
Alkalinity (mg CaCO3/L)	5	720	520	610	410	450	540	580
Salinity (mg/L)	10	37,000	39,000	44,000	42,000	42,000	38,000	31,000
Acetic Acid (mg/L)	1	66	39	69	29	67	33	<1
Propionic Acid (mg/L)	2	29	24	60	53	36	28	<2
TOC^*d*^ (mg/L)	1	96	64	100	73	97	56	< 1
Total Nitrogen (mg/L)	0.2	45	42	52	47	47	43	41
Total Phosphorus (mg/L)	0.01	0.11	0.38	0.12	0.25	0.06	0.15	0.10

*^*a*^Chromium, Copper, Nickel, Sulfur, NOx-N, Nitrate-N, Nitrite-N, and thiosulphate were not detected in any of the samples. ^*b*^LOR, Limit of Detection. ^*c*^TPH, Total Petroleum Hydrocarbons. ^*d*^TOC, Total Organic Carbon.*

**TABLE 2:** Chemical composition of deposits samples collected in the produced water system.

**Compound/element^a^**	**LOD^b^**	**DP1**	**DP2**	**DP3**
TPH^c^ C6–9 (mg/Kg)	0.2	5,900	22000	26000
TPH C10–14 (mg/Kg)	0.2	22,000	74000	87000
TPH C15–28 (mg/Kg)	0.4	1,400	83000	100000
TPH C29–36 (mg/Kg)	0.4	<0.4	8100	12000
TPH C > 36 (mg/Kg)	0.4	<0.4	3200	5300
Calcium (mg/Kg)	10	160	7100	1500
Chromium (mg/Kg)	1	11	120	77
Copper (mg/Kg)	1	7	1300	36
Iron (mg/Kg)	1	330,000	66000	58000
Potassium (mg/Kg)	10	<10	820	790
Magnesium (mg/Kg)	10	30	1100	970
Manganese (mg/Kg)	1	2,900	470	260
Sodium (mg/Kg)	10	<10	18000	36000
Nickel (mg/Kg)	1	8	260	170
Sulfur (mg/Kg)	10	77,000	41000	31000
Zinc (mg/Kg)	1	<1	680	1900
Total Nitrogen (mg/Kg)	10	430	13000	28000
Total Phosphorus (mg/Kg)	1	3	11000	11000
Ammonia-N (mg/Kg)	10	30	60	70
Chloride (mg/Kg)	10	1,700	6400	8000
Sulfate (mg/Kg)	10	40,000	10	10
Thiosulphate (mg/Kg)	2	600	82	140
Propionic Acid (mg/Kg)	2	120	<2	<2
Formic Acid (mg/Kg)	2	<2	30	70
TOC^d^ (mg/Kg)	0.1	10	14	19

*^*a*^NOx-N, Nitrate-N and Nitrite-N were not detected in any of the samples. ^*b*^LOR, Limit of Detection. ^*c*^TPH, Total Petroleum Hydrocarbons. ^*d*^TOC, Total Organic Carbon.*

### Microbial Enumeration

Serial dilution analysis indicated that microbial populations typically monitored by the oil and gas industry and previously associated with corrosion are present in all the sources evaluated in concentrations of 10–10^6^ Bact/mL-g. Sulfide producing microbes and APB were widespread across the oil production facility whereas iron utilizing microorganisms were present only in a few locations, as shown in Table 3. Stations treated with biocide (PW1 and PW2) reported similar levels of microorganisms than other stations without biocide treatment (PW3, PW4, and PW5). Microorganisms were also detected in the deposits samples despite the chemical treatment applied to the system. In fact, sample DP4 exhibited the highest concentration of sulfide producing and acid producing microorganisms of all samples collected.

**TABLE 3:** Enumeration of microorganisms associated to MIC corrosion.

**Sample**	**SPP^a^**	**APB^b^**	**IRB^c^**	**IOB^d^**
PW1	10^3^	10^2^	<10	<10
PW2	10^2^	10^2^	10^1^	10^3^
PW3	10^3^	10^2^	<10	<10
PW4	10^3^	10^2^	<10	<10
PW5	10^2^	10^1^	<10	<10
PW6	10^2^	10^2^	<10	<10
DP3	10^3^	10^1^	<10	<10
DP4	10^6^	10^3^	<10	<10
IW	10^2^	<10	<10	10^1^

*^*a*^SPP, Sulfide-producing prokaryotes; ^*b*^APB, Fermenting acid-producing bacteria; ^*c*^IRB, Iron reducing bacteria; ^*d*^IOB, Iron oxidizing bacteria.*

### Microbial Molecular Characterization

#### Characteristics of the 16S rRNA Datasets

A total of 3,991,895 (DNA-based) and 2,926,183 (RNA-based) sequence reads were obtained from the MiSeq sequencing. After removal of low-quality sequences, chimeras and singletons 3,536,010 (DNA-based) and 2,010,504 (RNA-based) high-quality sequences were used for the diversity profiling analysis. The number of sequences in each sampling location ranged from 34,532 to 203,508 DNA-based, and from 73,419 to 168,184 RNA-based. After rarefaction analysis with normalized sequences per sample (34,532), we obtained 493 zOTUs from DNA-based (ranged from 54 ± 2 to 285 ± 4 per sample) and 287 from RNA-based (ranged from 116 ± 5 to 198 ± 8 per sample). Reads and zOTU counts for the individual sample replicates are summarized in Supplementary Table S1. The Good’s coverage index of 0.99 (±0.005) for both sequencing approaches indicated that the datasets enclose all major microbial groups inhabiting the oil production facility (Supplementary Figure S1).

The shared microbial zOTUs analysis indicated that there are unique and shared zOTUs in each sample source (Supplementary Figure S2). Shared zOTUs between the three sources was 65, accounting for 78% of the total community in the injection water, 15% of deposits, and 17% of produced water. Most of the zOTUs were simultaneously detected in the produced water and deposits (263 zOTUs accounting for 53% of the total zOTUs detected). However, deposits hosted more individual species than other sample sources (105 zOTUs). Based on the Venn diagrams, unique zOTUs were also detected in the satellite stations and deposits when the same sample source was compared. Only 22% (82 zOTUs) of the total zOTUs detected in produced water were shared in all stations. Stations with biocide treatment (PW1 and PW2) exhibited more individual species than stations without biocide treatment. Similar to produced water, only 29% (125 zOTUs) of the total zOTUs detected in deposits were common in all samples. Shared zOTUs analysis between DNA and RNA-based profiling showed that most of the microorganisms present in produced water were detected with both sequencing approaches (325 zOTUs accounting for 82% of the total zOTUs detected in produced water). The detection of unique zOTUs with the DNA-based approach indicated that not all microorganisms in the system are active. Likewise, the detection of unique zOTUs with the RNA-based approach indicated that the system host rare taxa that are highly active.

#### Comparison of DNA and rRNA Amplicon Libraries

**FIGURE 2:**
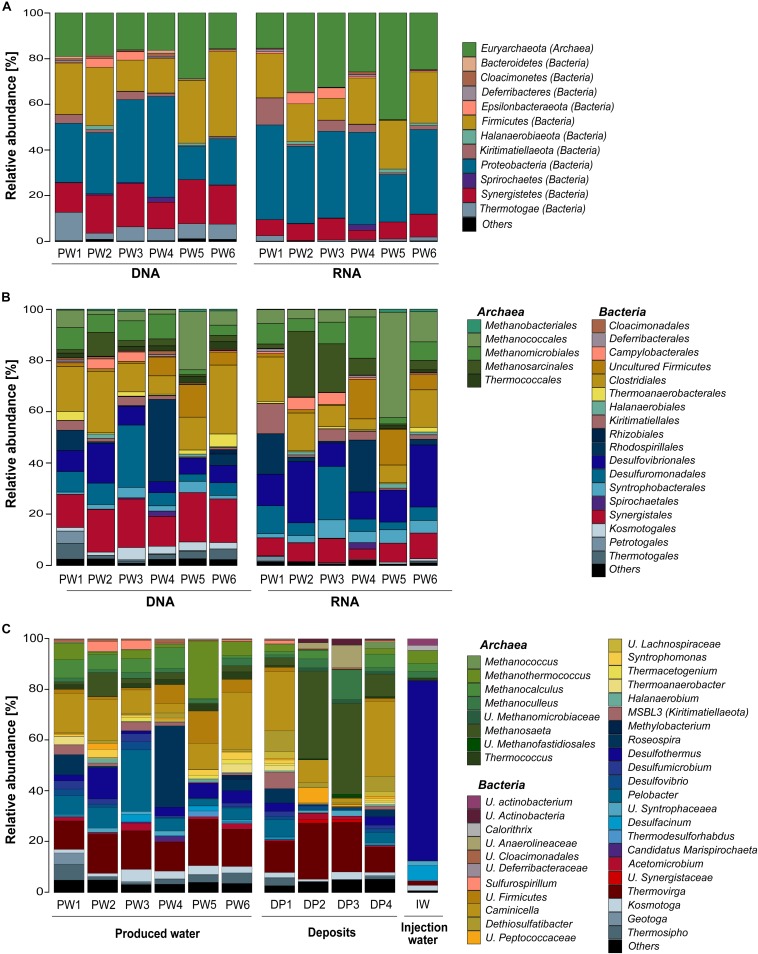
Microbial community composition in the oilfield retrieved from 16S rRNA gene and transcripts sequencing. **(A)** Total and active microbial communities in produced water samples at phylum level. **(B)** Total and active microbial communities in produced water samples at order level. **(C)** Total microbial community in produced water, deposits and injection water samples at genus level. U., unclassified. Phylogenetic groups accounting for <1% of all classified sequences were summarized in the artificial group “Others.” Results from the three replicates collected in each sampling location were pooled together.

Total and potentially active microbial communities in the produced water were dominated by Bacteria. Relative abundances of Archaea were higher in the RNA-based profiling than in the DNA-based profiling. Same dominant phyla were detected with both methodological approaches. However, the relative abundances of the phyla detected in each sampling location varied between DNA and RNA-based profiling (Figure 2A and Supplementary Table S2). The trend of variation in the abundance of each phylum was similar in all samples. Dominant phyla were *Proteobacteria* (28% DNA, 35% RNA), *Firmicutes* (24% DNA, 18% RNA), *Euryarchaeota* (19% DNA, 30% RNA), *Synergistetes* (16% DNA, 8% RNA), *Thermotogae* (7% DNA, 1% RNA), *Kiritimatiellaeota* (2% DNA, 4% RNA), and *Epsilonbacteraeota* (1% DNA, 2% RNA). At the order level, *Methanococcales, Methanosarcinales*, and *Desulfovibrionales* were more abundant in the RNA-based than in the DNA-based profiling (Figure 2B and Supplementary Table S3). *Clostridiales, Synergistales, Kosmotogales*, and *Thermotogales* orders showed the opposite trend. Dominant orders such as *Desulfuromonadales, Rhodospirillales*, and *Methanomicrobiales* presented similar abundances in the total and potentially active communities.

#### Microbial Community Composition of the Three Sample Sources at the Genus Level

DNA-based profiling of the abundant genera found in the different sampling locations of the oil production facility is shown in Figure 2C (complete list of genera is available in the Supplementary Table S4). Results indicated that produced water and deposits samples contained similar populations with differences in their abundance among samples. Results also evidenced that the microbial community existing in the injection water was markedly different from the other sample sources. An overview of the main microbial genera found in the production samples (water and deposits) allowed to differentiate two communities. The first type of community was evidenced in deposits samples DP2 and DP3. The community was principally composed by *Methanosaeta* ($\bar{\text{x}}$ 35%), *Thermovirga* ($\bar{\text{x}}$ 20%), *Methanoculleus* ($\bar{\text{x}}$ 8%), and *Caminicella* ($\bar{\text{x}}$ 6%) genera. The second type of community was found in all other samples, main species were *Caminicella* ($\bar{\text{x}}$ 16%), *Thermovirga* ($\bar{\text{x}}$ 14%), *Pelobacter* ($\bar{\text{x}}$ 8%), *Roseospira* ($\bar{\text{x}}$ 7%), *Methanothermococcus* ($\bar{\text{x}}$ 6%), *Desulfothermus* ($\bar{\text{x}}$ 5%), and *Methanocalculus* ($\bar{\text{x}}$ 5%). Differently, the microbial community in injection water was dominated *Desulfothermus* ($\bar{\text{x}}$ 71%), *Desulfacinum* ($\bar{\text{x}}$ 6%), *Methanothermococcus* ($\bar{\text{x}}$ 5%), *Methanocalculus* ($\bar{\text{x}}$ 3%), and *Methanoculleus* ($\bar{\text{x}}$ 3%).

#### Alpha Diversity Analysis of the Microbial Community

**FIGURE 3:**
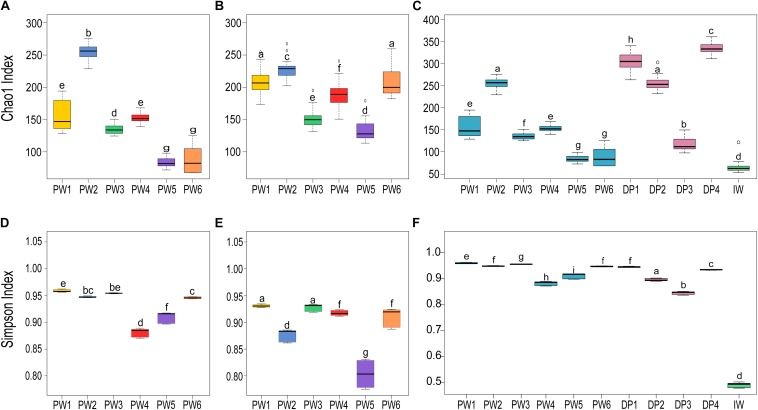
Alpha diversity indices of microbial communities in the different sampling locations. **(A,C,D,F)** Richness (Chao1) and diversity (Simpson) indices of the total communities. **(B,E)** Richness and diversity indices of the active communities. Boxes are extended from the 25–75th percentiles, the line in the box is plotted at the median. Whiskers represent the smallest and the largest value. ANOVA followed by Tukey’s multiple comparison tests was used to determine differences among stations. Samples with the same letter indicate that the diversity indices were not significantly different (*p* > 0.05).

Comparison of the biodiversity between total and potentially active communities in produced water samples showed significant (*p* ≤ 0.05, *t*-test) differences in the alpha diversity measurements. The richness index (Chao1) of the microbial community was highest for the RNA-based profiling and lowest for the DNA-based profiling (Figures 3A,B). Conversely, the diversity index (Simpson) showed the opposite pattern (Figures 3D,E). Statistical analysis of the alpha diversity also revealed significant differences in the biodiversity among sampling locations (*p* ≤ 0.05, ANOVA). Unexpectedly, stations with biocide treatment (PW1 and PW2) displayed higher richness than stations without biocide treatment under both sequencing approaches. A similar pattern was evidenced in diversity indices with the DNA-based analysis but slightly different from the RNA-based. The diversity of the potentially active community in station PW1 was the highest, whereas diversity in PW2 and PW5 were the lowest.

Alpha diversity metrics calculated from the DNA-based sequencing were significantly different (*p* ≤ 0.05, ANOVA) among sample sources and sampling locations (Figures 3C,F). In the comparison of sample sources, deposits presented the highest Chao1 richness, followed by produced water, and injection water. Simpson diversity measurements were similar between deposits and produced water, and lower in the injection water. Looking at the biodiversity among samples from the same source, both the samples from produced water and samples from deposits presented significant variations in the alpha diversity indices calculated.

#### Environmental Factors Affecting the Microbial Community Structure

**FIGURE 4:**
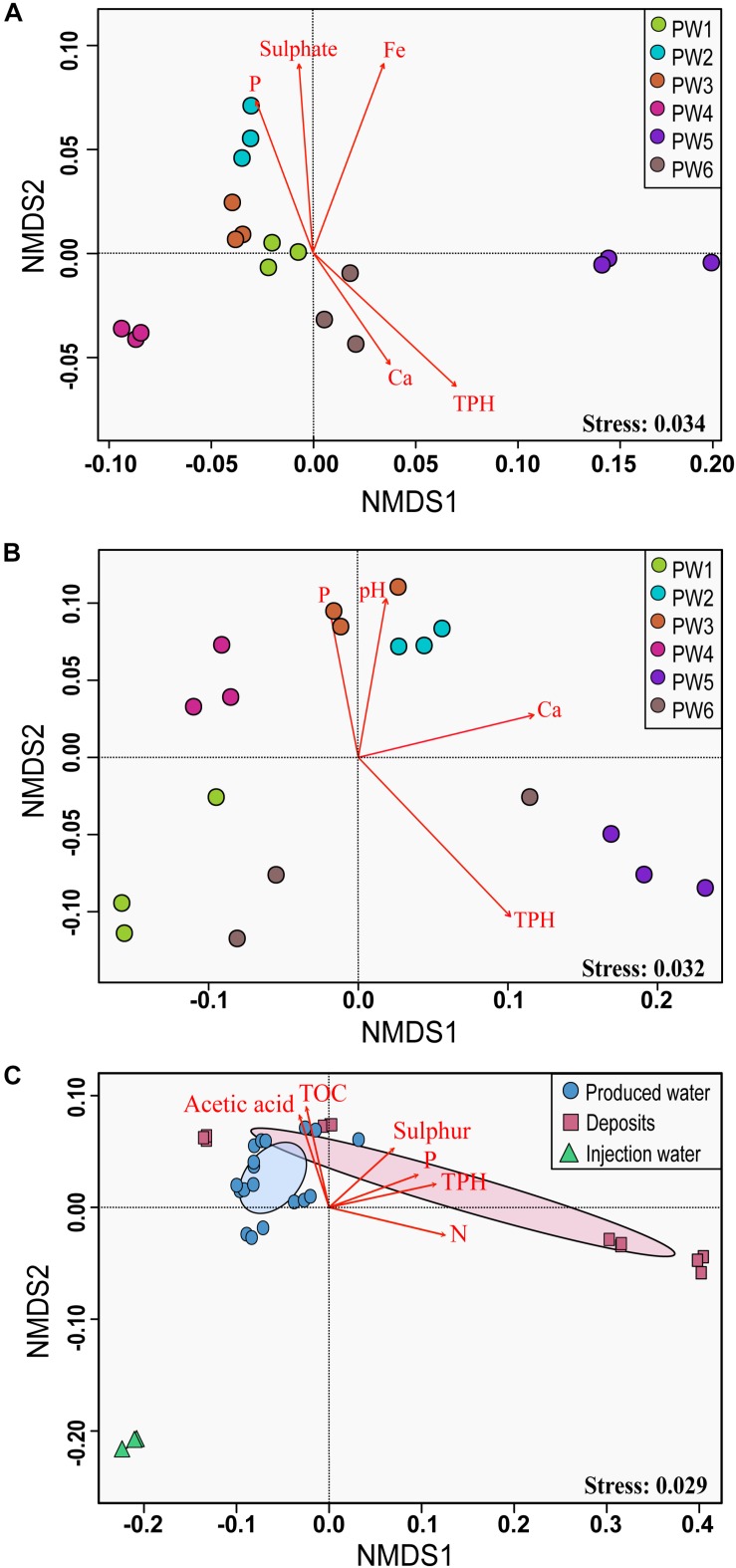
Non-metric multidimensional scaling (NMDS) analysis of the microbial communities. **(A)** NMDS for total microbial communities in produced water samples. **(B)** NMDS for active microbial communities in produced water samples. **(C)** NMDS for total microbial communities in all sampling locations. The analysis was based on weighted UniFrac distance matrices. Environmental parameters that were significantly correlated (*p* ≤ 0.05) to microbial community structure are indicated by arrows.

Non-metric multidimensional scaling ordination analysis showed differences in the microbial community structure of the produced water samples (Figures 4A,B). Greater differences were observed with the RNA-based sequencing approach, which showed a clear separation of the sampling points. The two-way PERMANOVA and two-way ANOSIM tests confirmed that the community structure between sequencing approaches, as well as among sampling locations had significant differences (*p* = 0.0001). We tested the correlation of physicochemical characteristics and fitted them onto the ordination to determine what properties were correlated to the total and active community composition. The structure of the total community (DNA based) was strongly influenced by concentrations of TPH (*p* = 0.001), iron (*p* = 0.001), sulfate (*p* = 0.006), and phosphorous (*p* = 0.008). On the other hand, the structure of the potentially active community (RNA based) was greatly influenced by the concentration of TPH and calcium (*p* = 0.002 and 0.007, respectively), followed by pH and phosphorous (*p* = 0.022 and 0.033, respectively).

Non-metric multidimensional scaling ordination with all sampling locations confirmed differences in the microbial community structure according to the sample source (Figure 4C). Injection water samples created a separated cluster away from deposits and produced water samples. According to the PERMANOVA and ANOSIM tests, differences were significative (*p* = 0.004 and *p* = 0.006, respectively). Pairwise comparison evidenced that the significant differences in the microbial structure were only related to the injection water samples. The comparison of the microbial structure in produced water and deposits samples showed no significant differences (*p* = 0.07). Correlation between environmental variables with the total microbial composition of all sampling points showed that concentration of TPH, nitrogen, phosphorous, TOC, sulfur and acetic acid impacted considerably the structure of the total community (*p* = 0.001, 0.001, 0.004, 0.015, 0.025, 0.031, respectively).

#### Association Networks of Specific Taxa With Sampling Location

**FIGURE 5:**
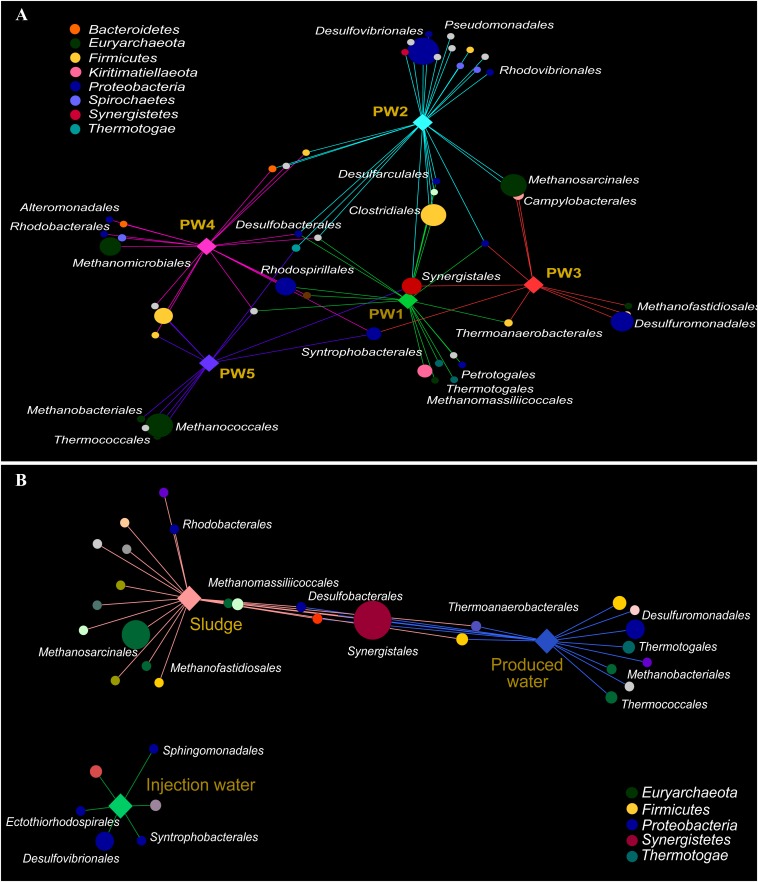
Correlation-based association network between microbial community (order level) and sampling location. **(A)** Association network of active community with produced water stations. **(B)** Association network of total community with sample sources. Only statistical significant microbial orders are visualized (*p* ≤ 0.05). The size of each node is proportional to the taxon relative abundance and the edge width corresponds to the association strength of each taxon with the sampling location. Color of nodes contributes to prominent microbial phyla. Hub nodes and edges are colored according to sampling location.

Association networks indicated that 85% of the total orders detected in the active community had significant biserial correlation coefficients with the sampling location (*p* ≤ 0.05). Most of the orders were associated with stations with biocide treatment PW1 and PW2 indicating that the environment at these locations favors the activity of several microorganisms (Figure 5A). The majority of the orders significantly associated with each station belong to the same microbial groups (sulfate reducing bacteria, fermenting bacteria, and methanogens).

The correlation-based association analysis of the orders significantly associated with the sample source (Figure 5B) was consistent with the multivariate analysis (Figure 4C). None of the orders that were significantly associated with the injection water was significantly associated with another sample source. The majority of the orders were significantly associated with only one source suggesting that the microbial community structure in the oilfield is driven by the specific conditions along the facility. Only 8% of the orders were significantly associated with both production sample sources.

#### Functional Profile Prediction

**FIGURE 6:**
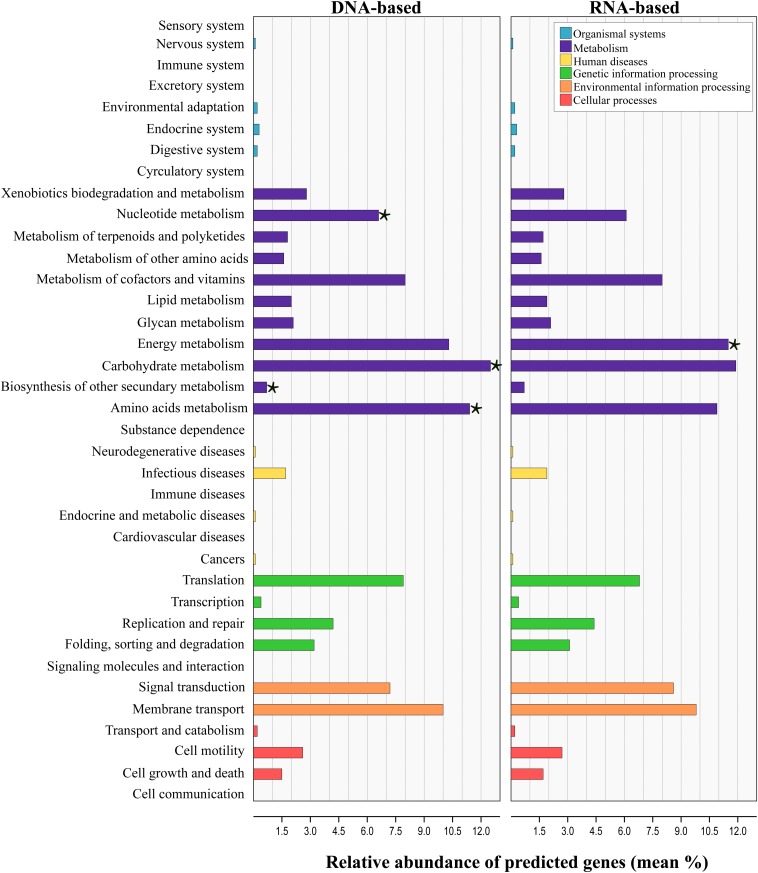
Prediction-based abundance of genes involved in KEGG level 2 categories in total and active communities in the produced water samples. Significant (*p* ≤ 0.05) biomarkers identified with the linear discriminant analysis (LDA) effect size are indicated by a star (⋆).

Tax4Fun analysis applied to infer the metagenomic content of the total and active communities in produced water predicted the presence of 6422 KEGG Orthologs (KO) across all samples (Supplementary Table S5). LEfSe analysis indicated that 43 from the 280 pathways found, were significantly different between communities recovered with both sequencing approaches (Supplementary Table S6). Level 2 KO predicted from the DNA and RNA-based profiling are presented in Figure 6. Overall, the functional structure of the communities was dominated by metabolism-related KEGG pathways, especially that of carbohydrates, amino acids, nucleotide, energy, cofactors and vitamins. Other dominant KEGG categories predicted were environmental and genetic information and processing, principally in pathways related to signal transduction, membrane transport, and translation. Genes related to cellular processes, human diseases and organismal systems were predicted with lower abundances. LEfSe analysis at this level showed that metabolic pathways related to carbohydrate, amino acids, nucleotide and biosynthesis of other secondary metabolites were biomarkers of the DNA-based analysis, whereas energy metabolism was a biomarker of the RNA-based analysis. Within the energy metabolism, the methane metabolism pathway was the most abundant.

Linear discriminant analysis test applied for the identification of the metabolic KEGG pathways associated with the sample source showed that 46 of the 139 pathways predicted were significantly differentially abundant among sources (Supplementary Table S7). According to the results, pathways related to the metabolism of amino acids and xenobiotics were significantly enriched in the deposits and production water samples, whereas pathways related to the energy and carbohydrate metabolism were enriched in injection water samples. It has to be noted, that the results from predicted functional profile based on 16S rRNA data can deviate from metagenomics profiling since taxonomic identification does not necessarily relates to the presence of functional genes. Additionally, zOTUs derived from unknown taxa limit this prediction. In this sense, the predicted metabolic pathways identified here remain to be validated by metagenomics studies in future.

## Discussion

### DNA Versus RNA-Based Diversity Profiling

The comparison between the DNA and RNA-based diversity profiles indicated that not all the microbial community members were active and that not all the active members were detected with the DNA approach. Predominant orders in the community were recovered with both methodologies, but significant differences in their relative abundance were evidenced. One of the main differences detected was the reduction in the abundances of all thermophilic orders (*Thermotogales, Thermococcales, Thermoanaerobacterales, Synergistal*es, *Petrotogales*, and *Kosmotogales*) in the RNA approach. The temperature at the time of sampling in all analyzed locations was approximately 40°C, which could explain the lower abundances of thermophiles in the active communities. Thermophilic microorganisms are commonly associated with native populations in oil reservoirs (Ollivier and Magot, 2005; Song et al., 2017), however, temperature gradients generated during the production process favor the growth of mesophilic microorganisms at lower temperatures (Li et al., 2017b). The effect of the temperature variations on the community structure of active populations has been previously evaluated (Salgar-Chaparro et al., 2019). The authors pointed out that temperature has a significant impact in shaping the microbial composition of oilfield systems which, in turns, affects its corrosivity. Other microbial orders that exhibited a decrease in the relative abundances with the RNA-based approach were *Clostridiales, Rhodospirillales*, and *Synergistal*es. Several species that belong to these taxonomic groups have been associated with oil degradation, which is one of the principal metabolisms in oil reservoirs along with fermentation, methanogenesis and sulfate reduction. It has been reported that oil-degrading microorganisms need to be in contact with the petroleum hydrocarbons to be able to use them as electron donors, while also being in contact with the water phase for reaching electron acceptors such as sulfate, nitrate, or ferric iron (Pannekens et al., 2019). Therefore, considering that the water cut in all sampling locations was close to 95%, it is inferred that the oil fraction in the fluid could have been a limiting factor for the microbial growth of these populations, which might provide an explanation to their lower abundances in the RNA-based approach compared to the DNA-based approach. Conversely, sulfate reducers like *Desulfuromonadales, Desulfovibrionales*, and *Syntrophobacterales*, as well as, methanogens such as *Methanobacteriales, Methanococcales, Methanomicrobiales*, and *Methanosarcinales* showed an increase in their abundance in the active communities. Higher abundance of these populations in the RNA-based analysis suggests that the oil production facility provides suitable conditions for the metabolic activities of these particular groups.

Alpha and beta diversity analysis of the total and active populations confirmed that differences in communities recovered with both sequencing approaches were significant. Higher diversity values obtained with the DNA-based analysis are the result of recovering active, dormant and dead cells when studying the DNA molecule. Conversely, RNA-based analysis only retrieves information about the active cells, thereby lower values of biodiversity are often obtained. Similar results have been described in other studies where a comparison of the DNA and RNA sequencing profile was carried out (Angel et al., 2013; Kim et al., 2013; De Vrieze et al., 2016; Inkinen et al., 2016; Li R. et al., 2017). Dissimilarities in the diversity profile of total and active communities were also reflected in the predicted functional capability. Fundamentally, genes involved in energy metabolism, principally the genes related to methane metabolism were significantly enriched in the RNA-based sequencing approach. Higher capability for using methane pathways resulted from the increase in the relative abundance of methanogens in the active communities.

Differences between the DNA and RNA-based results highlighted the importance of using a complementary methodological approach for studying microbial populations. It has to be noted that RNA-based methods have disadvantages linked to more laborious extraction procedures, the susceptibility of RNA to degradation, presence of multiple copies of ribosomes per cell, and the existence of rRNA reserves in dormant cells (Angel et al., 2013). Even considering these drawbacks, RNA-based methods can better reflect the active members of oilfield communities compared to DNA surveys, which is essential for assessing the potential involvement of microorganisms in corrosion since only metabolically active microorganisms can cause MIC. Identification of active microorganisms in oilfield systems also provides relevant information with regards to the efficacy of biocide treatments. The detection of active microorganisms after exposure to biocide treatments indicates inefficiency of treatments or inadequate treatment dosages, which can lead to the emergence of resistant communities. Therefore, early detection of microbial activity in industrial facilities would help optimize mitigation strategies to control MIC.

### Microbiological Assessment in the Oil Production Facility

The microbial community recovered from the water and deposits samples provided a general representation of the planktonic and sessile populations inhabiting the production system. Microbiological characterization using the complementary approach indicated that total and active microbial communities in the oilfield were dominated by bacteria in a proportion that ranged from 50 to 80%. Taxonomic identification of the sequencing reads revealed that several of the microorganisms present in the oil facility have been previously reported in other oil production facilities and corrosive environments.

Main corrosion damages in the oil facility have been reported downstream satellite stations and water-oil separation. As mentioned before, systems with major corrosion problems are under chemical treatment with biocides (THPS or Acrolein) to reduce the risk of MIC. Nonetheless, produced water samples with THPS treatment showed similar microbial composition to the samples without biocide treatment. Likewise, microbial enumeration showed a similar concentration of microorganisms in treated and non-treated stations. In the same way, deposits samples that are treated with Acrolein reported the same or greater microbial concentrations than produced water. All these findings suggest that the biocide treatments, which are intended to inactivate all microorganisms present, are not effective against microorganisms in the facility. THPS biocide is widely used in the industry due to its adequate characteristics such as low toxicity, broad-spectrum activity and ability to dissolve ferrous sulfide deposits (Conlette, 2014). However, it is known that the persistent use of the same biocide chemical will lead to the selection of resistant microorganisms over time (Li et al., 2016). Moreover, biocide chemicals can adsorb onto deposits, which will result in underdosing of chemical treatments, therefore reducing the biocide residual concentration required to maintain microbiological control. Likewise, there are microorganisms that can degrade THPS at sub-lethal concentrations, using it as a nutrient for growth (Vorholt et al., 2000). THPS degradation produces formaldehyde under aerobic conditions and methanol under anaerobic conditions (Sharma et al., 2018). Since oil production systems are anaerobic environments, methanol is the most probable byproduct in THPS degradation. This molecule can be used as a carbon source by several of the methanogens detected in the oilfield. Additionally, THPS dissociation releases sulfate and phosphorus to the environment, both also used by many microorganisms in their metabolic functions (Sharma et al., 2018). In fact, station PW2 that undergoes biocide treatment had a higher phosphate concentration compared to other stations and was the sole station where sulfate was detected. It can be speculated that the presence of these additional components in the produced water treated with THPS is associated with the higher richness values measured compared to stations without chemical treatment.

Microbiological analysis of the deposits samples recovered from different locations in the produced water pipework showed significant variations in the community structure among samples. Differences were also detected in the chemical composition of the deposits, particularly relating TPH, iron, sulfur, and sulfate concentrations. It is known that a non-homogeneous environment generates microniches with dominant populations adapted to the local conditions (Korona, 1996), which could explain the variability in the microbial communities recovered from the same system. Compared to produced water samples, deposits samples presented a higher number of zOTUs and richness values. More zOTUs in the deposits may be related to species accumulation over time in the biofilm communities living in the deposits, whereas water samples only reflect the community in the fluid at the time of sampling. In addition, other studies have shown that extracellular DNA (eDNA) can potentially be adsorbed in deposits or surfaces over time. eDNA can remain in the environment as part of sediment particles that can preserve it from degradation (Dell’anno and Corinaldesi, 2004; Torti et al., 2015; Corinaldesi et al., 2018). Indeed, corrosion products have been nominated as a repository of eDNA in the Makama et al. (2018) investigation, who found eDNA in biofilm-free corroded surfaces. Due to the difficulty of extracting good quality RNA from the deposits samples, it was not possible to determine if the higher richness detected in these samples was a consequence of recovering eDNA preserved in the schmoo. According to the chemical composition, deposits had high levels of metals which correlates with active corrosion evidenced in the produced water recovery system. Likewise, high levels of total sulfur and sulfur compounds including sulfate, thiosulphate were detected in deposits. The source of these sulfur compounds remain unclear since both produced and injection waters did not have considerable concentrations of such compounds (<10 ppm of sulfate/thiosulphate). It is plausible to expect that small amounts of those compounds can also be adsorbed and accumulate in deposits over time (Suarez et al., 2019b). It has been documented that high doses of THPS can lead to the precipitation of the sulfate introduced by the THPS, which cause scale formation downhole (Li et al., 2016). The presence of these additional nutrients in the deposits is expected to attract more species and result in higher richness, as evidenced in this study.

As mentioned before, the production facility has been flooded for over 30 years to stimulate the reservoir and increase oil recovery. Long-term water injection can modify the indigenous microbial community structure in oil systems (Zhang et al., 2012; Lenchi et al., 2013; Gao P. et al., 2015). Apart from the microbiological contamination, water injection is problematic to oil reservoirs by providing nutrients and electron acceptors to the resident microorganisms (Magot et al., 2000; Varjani and Gnansounou, 2017). For evaluating the impact of this practice in the indigenous oilfield microbial community, and determining if the populations detected were the result of microbial contamination during water injection, the source water used in the injection system was also characterized. Microbiological analysis showed a clear difference in the microbial structure between production water and injection water which suggests that the secondary recovery practices have not had a significant impact on the community widespread in the facility. Moreover, species association analysis confirmed that none of the orders found in the injection water sample was significantly associated with the produced water or the deposits samples. Similarly, chemical analysis of the samples showed that source water contains lower levels of ions, metals and organics compared to produced water; therefore, it is not providing additional nutrients to the community in the reservoir. These results are in disagreement with similar investigations in other oilfields, where significant changes in the microbial structure of the oil reservoir were evidenced after the water injection (Gao P. et al., 2015; Gao P. K. et al., 2015). The likely explanation of this phenomenon is that the ratio of an external source for water injection is only 5:95 source water:produced water. The above indicates that potentially corrosive microbial populations found in the oil production facility are likely coming directly from the reservoir.

Dominant microorganisms found active in the facility belong to three microbial groups, sulfate reducers, fermenters, and methanogens; all of these previously associated with MIC processes (Machuca et al., 2017; Machuca and Polomka, 2018; Vigneron et al., 2018; Suarez et al., 2019a). Microbial interactions among these populations have been previously studied (Raskin et al., 1996; Morris et al., 2013; Ozuolmez et al., 2015; Sela-Adler et al., 2017). Syntrophic interactions between fermenters (H_2_ producing microbes) and methanogens (H_2_ consuming microbes) are widely known. Due to the physiological versatility of several sulfate reducers, similar interactions can occur between fermenters and sulfate reducers, which can use the H_2_ produced by fermenters as electron donor in respiration, or methanogens and sulfate reducers, which can also have the capability to obtain energy from fermentation providing nutrients such as H_2_ and acetate to methanogenic species (Morris et al., 2013). Co-existence of methanogens and sulfate reducers is usually reported in oil production facilities (Lenchi et al., 2013; Lin et al., 2014; Varjani and Gnansounou, 2017), however, microbial interactions appear to be affected by the presence of sulfate (Oremland and Polcin, 1982; Okoro and Amund, 2018). In the presence of high sulfate concentrations, sulfate reducers compete with methanogens for the availability of nutrients. Conversely, low sulfate content environments, such as the produced water studied here, favor syntrophic interactions (Paulo et al., 2015). Thus, the increase in the relative abundances of methanogens and sulfate reducers in the active communities might be related to a symbiotic relationship.

Multispecies biofilms formed by same microbial groups found in this investigation have been reported to accelerate UDC of steel (Larsen and Hilbert, 2014; Machuca et al., 2017; Shukla and Naraian, 2017; Suarez et al., 2019a). It is known that different interactions between microorganisms might induce a cascade of biochemical reactions that cause more severe corrosion than single-species biofilms (Kip and Van Veen, 2015). In the presence of deposits, microbial cells are attracted to these particles as they provide nutrients, a suitable environment for the synergistic interactions of sessile communities, and also protection from shear forces and biocides (Kagarise et al., 2017). Microbial communities can interact with the deposits changing their properties, such as making them more electroactive or precipitating new corrosive species in the metal surface, which favors the formation of microenvironments and differential cells that can cause localized corrosion (Machuca et al., 2011). The existence of a schmoo layer previously suggested that corrosion is taking place in the oilfield assets by UDC mechanisms. Nonetheless, the detection of active microbial populations with reported corrosive metabolisms in this investigation provided more evidence to the hypothesis that the corrosion processes in the facility may be the result of UDC enhanced by MIC mechanisms. Further laboratory investigations simulating the oilfield conditions are required to study the possible MIC-UDC mechanisms involved.

## Conclusion

Complementary analysis of DNA and RNA-based amplicon sequencing allowed to assess differences in the microbial composition of total and active communities in the oil facility. It was demonstrated that DNA results alone could lead to underestimation of active members in the community. By implementing the RNA-based sequencing, it was found that not all microorganisms in the communities were active whereas other community members showed an increase in their relative abundances, which is proposed to be related to higher activity. A better characterization of active microorganisms can improve the understanding, mitigation and prediction of MIC processes. Moreover, this methodological approach can be used to evaluate the impact that operational conditions like temperature and water chemistry have on microbial activity and community structure. The reduction on the relative abundances of thermophilic species in the active community seen in this study was likely to be related to the decrease in temperature from the reservoir to the oil production facility. In addition, this study showed the detection of active microorganisms at biocide treated locations, which added to the identification of similar microbial composition and cells concentration in locations without biocide treatment suggesting poor efficacy of the mitigation treatments. Bias in the DNA-based analysis resulted in an underestimation of the predicted capability of the community for using methane pathway in the energy metabolism, which was correlated with the lower abundance of the methanogenic microorganisms in the total community. The detection of active microorganisms with reported corrosive metabolisms provided more evidence that microorganisms might have been involved in the localized corrosion detected in oil production assets.

## Data Availability Statement

The datasets generated for this study can be found in the National Centre for Biotechnology Information (NCBI), under the accession number PRJNA525758 (http://www.ncbi.nlm.nih.gov/bioproject/525758).

## Author Contributions

SS-C and LM designed the experiments. SS-C conducted the sampling, executed the experiments, carried out the microbial analysis, and prepared the manuscript with the contribution of LM.

## Conflict of Interest

The authors declare that the research was conducted in the absence of any commercial or financial relationships that could be construed as a potential conflict of interest.
