# Supplementary material for: Complementary DNA/RNA-Based Profiling: Characterization of Corrosive Microbial Communities and Their Functional Profiles in an Oil Production Facility
Source: Front Microbiol. 2019 Nov 7;10:2587. doi: 10.3389/fmicb.2019.02587 (PMC6853844; doi:10.3389/fmicb.2019.02587)
Supplement: Supplementary file 8 [file Image_1.pdf]

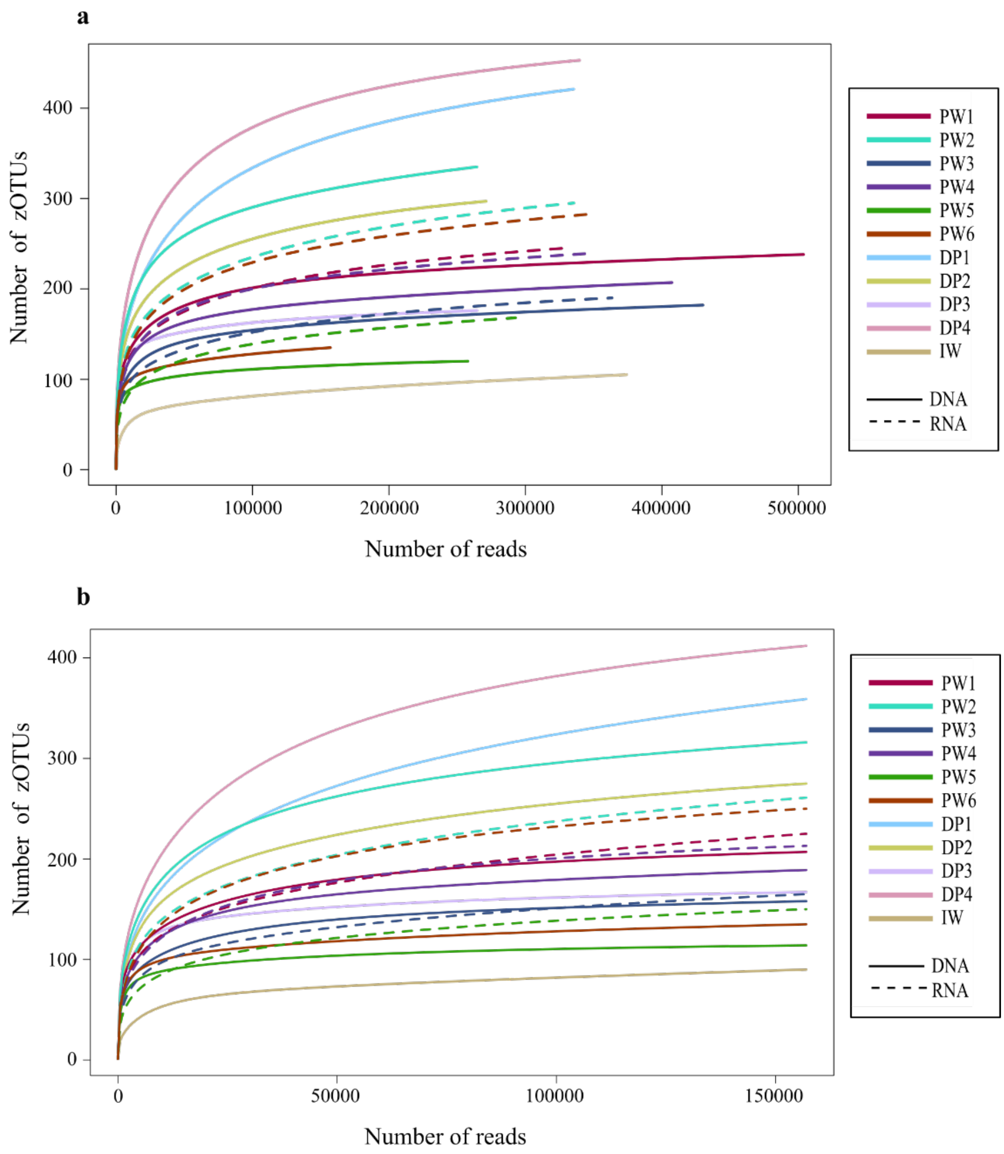

Supplementary Figure 1. Rarefaction curves constructed with the reads retrieved from DNA and RNA based sequencing of the 16S rRNA gene. a) Rarefaction calculated with the complete set of reads per sample, b) Rarefaction calculated for a subset of 157000 reads per sample.
