# Supplementary material for: Complementary DNA/RNA-Based Profiling: Characterization of Corrosive Microbial Communities and Their Functional Profiles in an Oil Production Facility
Source: Front Microbiol. 2019 Nov 7;10:2587. doi: 10.3389/fmicb.2019.02587 (PMC6853844; doi:10.3389/fmicb.2019.02587)
Supplement: Supplementary file 9 [file Image_2.pdf]

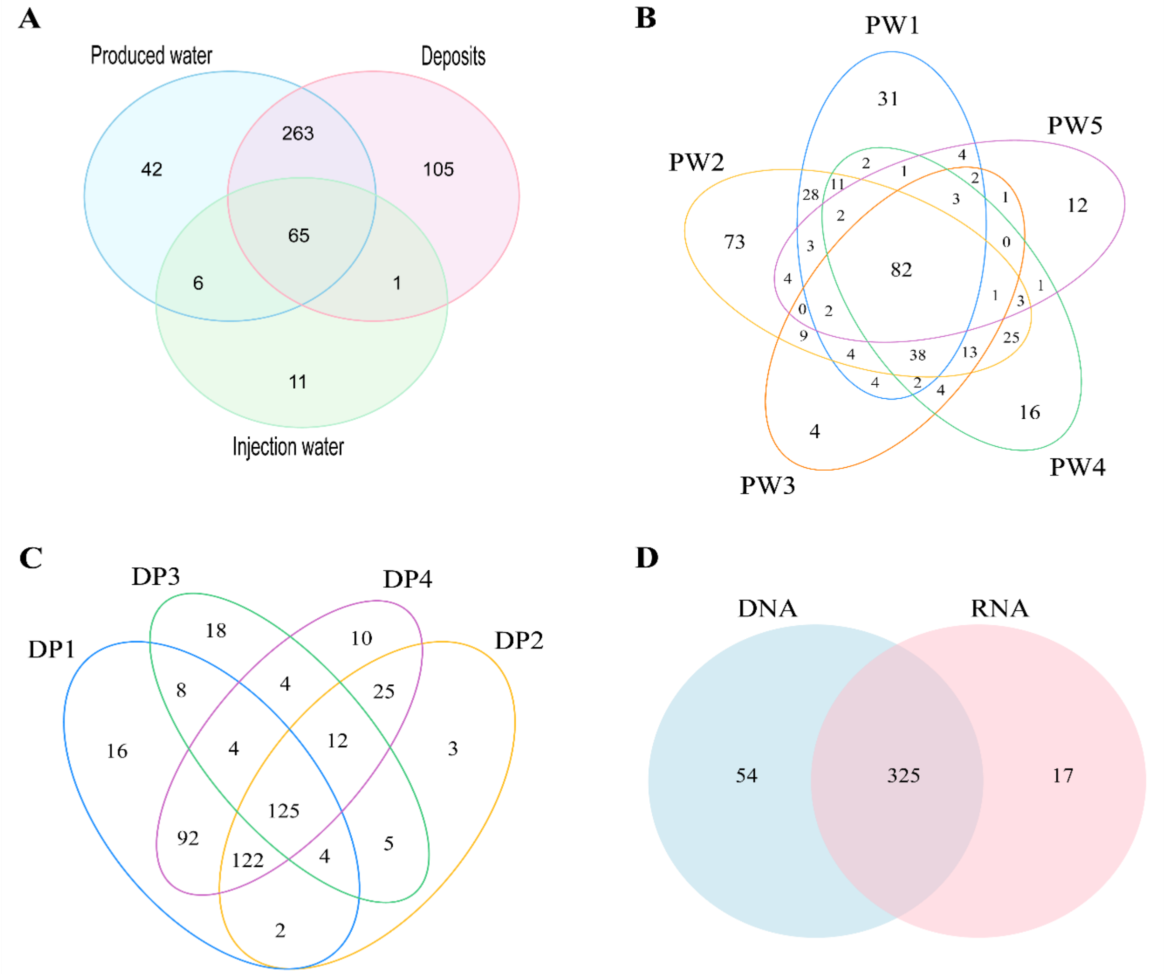

Supplementary Figure S2. Venn diagrams showing shared and unshared operational taxonomic units (zOTUs) among sample sources (A), satellite stations (B), deposits samples (C), and DNA-RNA based profiling analysis of produced water (D).
